# A Case of Presentation of the Axilla at Full Term: Spontaneous Delivery of the Fœtus

**Published:** 1901-06

**Authors:** F. Percy Elliott

**Affiliations:** Medical Officer to the Walthamstow Dispensary.


### A CASE OF PRESENTATION OF THE AXILLA AT FULL TERM: SPONTANEOUS DELIVERY OF THE FŒTUS.

BY

F. Percy Elliott, M.B., C.M. (Aberd.),

*Medical Officer to the Walthamstow Dispensary.*

---

Mrs. H., æt. 35 years, seventh labour. With the exception of the first the presentation at each labour had been abnormal. On the last occasion the presentation was one of shoulder, with prolapsed funis. The membranes ruptured early, and version was performed under chloroform. It was noted then that the pelvis was particularly roomy. Beyond this there was no abnormality made out. The present labour commenced on February 7th, the membranes rupturing at 2 p.m. The patient was seen at 5 p.m. on the same day, at which time the os was not sufficiently dilated to allow of the presentation being diagnosed. Abdominal palpation suggested a transverse presentation. Labour pains did not begin until 2 a.m. on the following day, the liquor amnii escaping freely in the meantime. The patient was seen for the second time at 9 a.m. on this day, when the left axilla was found presenting well into the vagina. The pains being frequent and strong, the presenting part so low down, and bearing in mind the roomy size of the pelvis, it was decided to leave delivery to nature, for a time at any rate.

Shortly after my arrival the left axilla appeared at the vaginal outlet, and following this came the head extended with the occiput lying on the right lumbar region of the fœtus, the left arm displaced dorsally and the right across the neck, the rest of the fœtus coming in the usual way.

The time occupied for delivery of the fœtus after labour pains commenced was only seven hours, while less than half an hour elapsed after the axilla and shoulder appeared at the vaginal outlet. The child, a stillborn one, was very large, weighing nearly 7 lbs. Its left axilla and that side of the chest was deeply ecchymosed, and the head and neck very livid. No fracture of any bones could be detected.

There was no laceration of any part of the maternal genital tract, and recovery was uninterrupted.

This case, besides recording a rare presentation, is illustrative of the marvellous power of nature in overcoming what would appear an impossible task. The pelvis, it is true, was capacious, but the fœtus also was large.

